# Pre-emptive pharmacogenetic testing in Italy: a review of evidence and multidisciplinary consensus on key priorities for implementation

**DOI:** 10.3389/fpubh.2026.1763975

**Published:** 2026-03-12

**Authors:** Angelica Valz Gris, Antonio Cristiano, Francesco Di Berardino, Erika Giacobini, Vittoria Tricomi, Angelo Maria Pezzullo, Erika Cecchin, Valeria Conti, Amelia Filippelli, Fiorella Gurrieri, Giovanna Liuzzo, Stefania Boccia

**Affiliations:** 1 Section of Hygiene, Department of Life Sciences and Public Health, Università Cattolica del Sacro Cuore, Rome, Italy; 2 Experimental and Clinical Pharmacology Unit, Centro di Riferimento Oncologico di Aviano, IRCCS, Aviano, Italy; 3 Department of Medicine, Surgery, and Dentistry, University of Salerno, Baronissi, Italy; 4 Clinical Pharmacology Unit, University Hospital “San Giovanni di Dio e Ruggi d’Aragona”, Salerno, Italy; 5 Department of Medicine and Surgery, Universitá Campus Bio-Medico Di Roma, Rome, Italy; 6 Operative Research Unit of Medical Genetics, Fondazione Policlinico Universitario Campus Bio-Medico, Rome, Italy; 7 Department of Cardiovascular Sciences, Fondazione Policlinico Universitario A. Gemelli-IRCCS, Rome, Italy; 8 Department of Cardiovascular and Pulmonary Sciences, Catholic University School of Medicine, Rome, Italy; 9 Department of Woman and Child Health, Fondazione Policlinico Universitario A. Gemelli IRCCS, Rome, Italy

**Keywords:** adverse drug reaction, genetic test, implementation science, personalized prevention, pharmacogenetic

## Abstract

**Background:**

Pre-emptive pharmacogenetic (PGx) testing involves identifying genetic variants associated with drug response before prescribing medication, with the aim of guiding drug selection or dosing to reduce adverse reactions and improve outcomes. Despite decreasing costs and the growing feasibility of multi-gene panels, implementation of pre-emptive PGx testing remains limited in Italy. This study aimed to evaluate the potential impact of pre-emptive PGx testing in Italy and to identify barriers and strategies for its implementation.

**Methods:**

We conducted a review of current evidence and a multistage consultation process with Italian experts from pharmacology, laboratory medicine, genetics, clinical care, and public health.

**Results:**

Our analysis supports the clinical utility, economic sustainability, and feasibility of pre-emptive PGx testing. However, significant barriers persist, including limited real-world data, unclear reimbursement mechanisms, insufficient laboratory and IT infrastructure, inadequate clinician training, and patient concerns related to privacy and data protection.

**Conclusion:**

To enable broader implementation, strategic actions are needed across six areas: regulatory alignment, research investment, professional training and result interpretation, public awareness and consent, laboratory infrastructure, and IT systems and data governance.

## Highlights

- Pharmacogenetic testing has been associated with reductions in adverse drug reactions and improved optimization of drug therapy; however, in Italy the uptake of pre-emptive testing remains limited and heterogeneous, with persistent organizational, regulatory, and infrastructural constraints.
- This study provides an integrated assessment of the potential impact and implementation barriers for pre-emptive pharmacogenetic testing in Italy, combining evidence from the literature with structured input from domain experts.
- The analysis synthesizes considerations on clinical utility, economic sustainability, and patient acceptability, and identifies continuing deficiencies in infrastructure capacity, reimbursement arrangements, and clinician training as major determinants of the current implementation gap.
- Achieving effective and equitable integration will likely require updates to national guidance, clearer reimbursement mechanisms, strengthening of laboratory and digital infrastructures, and expansion of clinician training, supported by targeted and sustai ned investment within the Italian National Health System.

## 1 Introduction

Pharmacogenetics (PGx) is the study of how inherited genetic variation influences an individual’s response to medications (1). ADRs represent a significant public health concern, contributing to 0.5–12.8% of hospital admissions in Europe and imposing substantial healthcare costs (2). Evidence from multiple studies links ADR occurrence to genetic markers relevant to drug metabolism and response. In one assessment of spontaneous reports conducted in the United Kingdom, about 9% of adverse drug reports were associated with medications for which pharmacogenomic prescribing guidance could be used to mitigate ADR risk (3). Beyond improving drug safety, PGx holds great potential for optimizing drug efficacy and dosing (1, 4, 5).

In the past 20 years, the increasing availability of genetic data, driven by large-scale biobanks and national genome initiatives and a growing interest of drug regulatory bodies and health economics authorities, has led to a rise in studies exploring the associations between genetic variants and drug metabolism, with an expanding body of evidence supporting the role of PGx in personalized medicine (1, 4). In addition, the decreasing cost and improved feasibility of multi-gene panels have highlighted the potential for broader application of PGx, emphasizing the possible impact of expanding its use through pre-emptive genotyping strategies (6). Pre-emptive PGx is defined as the systematic testing of patients for actionable genetic variants before any drug therapy prescription, with the goal of using this information to guide potential future drug selection or dosing to prevent ADRs and optimize therapeutic efficacy (7, 8). Despite the potential clinical benefit of pre-emptive PGx, the implementation in European health care systems remains limited due to criticisms in assessing the clinical utility of PGx-assisted treatment strategies and organizational challenges related to their integration into the routine clinical workflow (1, 9–12).

One of the most significant challenges in the implementation of PGx is the limited number of well-established gene-drug pairs that provide clear, evidence-based guidelines for modifying therapeutic regimens (13, 14). While numerous genetic variants associated with drug response have been identified, their clinical utility remains under investigation. Furthermore, there is a considerable debate regarding what constitutes sufficient clinical evidence to demonstrate the utility of PGx testing due to the heterogeneity of the drugs and outcomes to consider, and the actual evidence needed (9, 14, 15). This uncertainty complicates the development of standardized guidelines and hinders its widespread adoption in clinical practice. Additionally, logistical barriers impede PGx integration into healthcare systems, including reimbursement constraints, inadequate laboratory infrastructure and workforce training, and challenges in integrating genetic data with clinical informatics systems (10–12). As a result, despite the growing interest in PGx, these challenges have resulted in an inconsistent and heterogeneous use of pharmacogenomic tests worldwide.

In Italy, this situation has led to the emergence of research and hospital centers that have initiated early-stage implementations of preventive strategies based on multi-gene panels for relatively large populations, alongside centers where even widely accepted tests, such as the *DPYD* test for personalizing therapy of many solid tumors, remain underutilized (16, 17). Similar situations are found in many other European countries (18). This inconsistency reflects the ongoing challenges in achieving a coordinated, regulated approach to PGx, hindering its broader adoption and full integration into clinical practice.

This paper examines the potential impact and key barriers to implementing pre-emptive PGx testing in Italy, assessing the main opportunities and challenges involved and proposing strategic actions to support its effective and sustainable adoption.

## 2 Methods

This study was part of the European project “A PeRsOnalized Prevention roadmap for the future HEalThcare” (PROPHET), which aims to develop a Strategic Research and Innovation Agenda (SRIA) to support the implementation of effective personalized programs to prevent chronic diseases (19). A key task of the project is evaluating the potential for implementation of personalized prevention strategies across different national contexts. The assessment of pre-emptive PGx testing was considered highly relevant by the PROPHET consortium, given its potential impact and the shared challenges and stages of implementation observed across European countries.

In June 2024, a technical group comprising researchers of the Università Cattolica del Sacro Cuore and a multidisciplinary Steering Committee (SC), including Italian experts from various fields related to PGx implementation, were assembled. The SC included pharmacologists, laboratory expert, geneticists, public health experts, as well as clinicians from relevant specialties such as anesthesiology, cardiology, and oncology.

**Figure 1:**
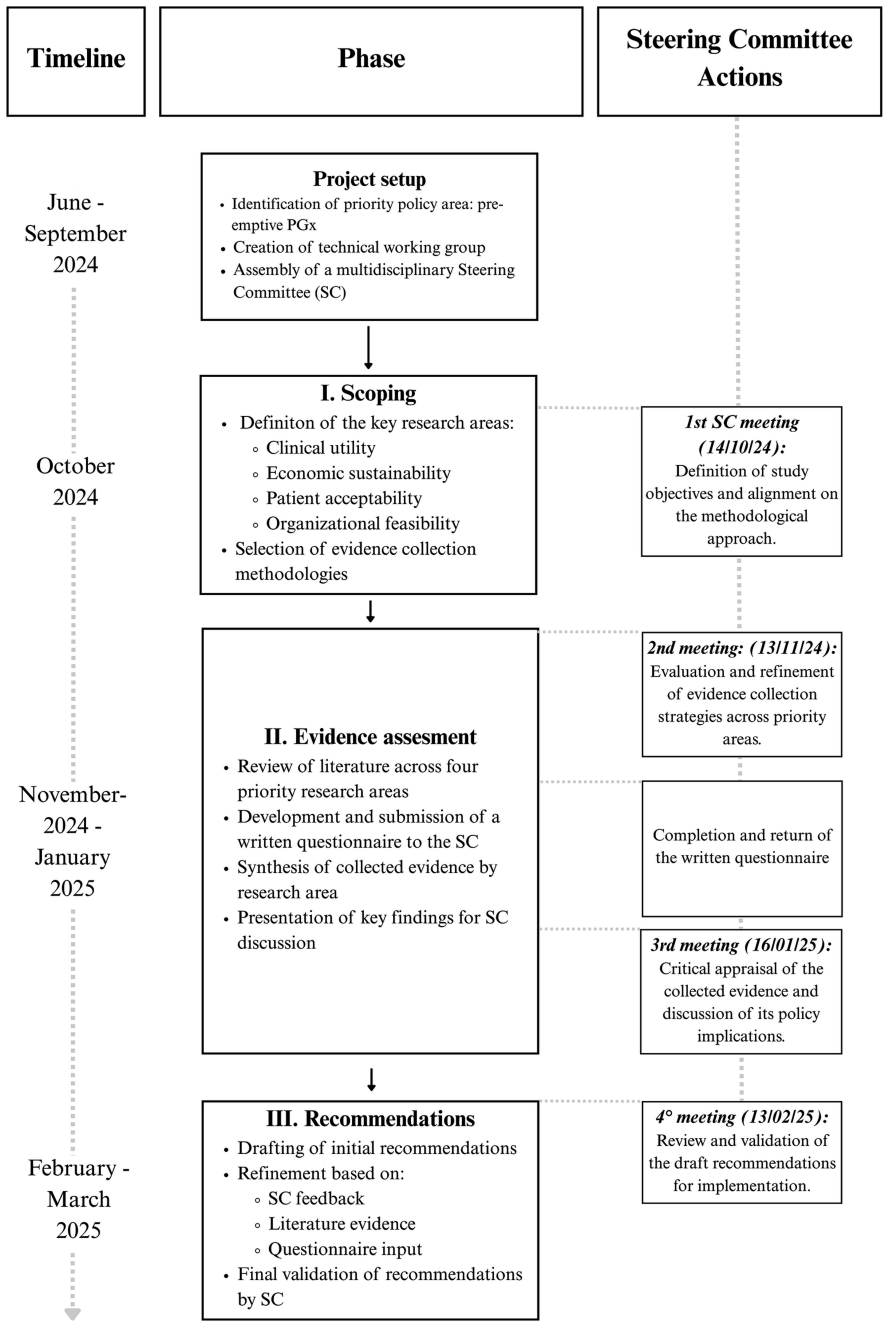
Methodological framework and timeline for the evaluation of pre-emptive pharmacogenetic testing in Italy.

The study was designed in three phases: *Scoping*, *Evidence Assessment,* and *Recommendations*, each of which involved discussion and validation by the SC (Figure 1).

### 2.1 Scoping

The first phase aimed at contextualizing the issue and identifying key research areas to explore to gather the necessary elements for evaluating both the potential impact and barriers. During this phase, the SC was also assembled. Experts were selected to ensure representation across the main disciplines relevant to pre-emptive PGx implementation, including four experts in pharmacology, two in laboratory medicine, one in medical genetics, two in public health, and clinicians from psychiatry, cardiology, oncology, primary care and clinical biochemist (one expert from each area). Through this first consultation with the SC, the priority research areas to be developed in the subsequent Evidence Assessment phase were identified.

### 2.2 Evidence assessment

The technical group collected evidence across the four research areas prioritized during the Scoping phase. The four key research areas were: clinical utility, economic sustainability, patient acceptability, and organizational feasibility. Narrative literature reviews were conducted for each research area, using PubMed as the primary source of peer-reviewed evidence. Search strategies were tailored specifically to each topic. For clinical utility, evidence from meta-analysis and randomized controlled trials (RCTs) were collected. An analysis of the guidelines issued by the major pharmacogenomics professional societies was also conducted. Literature addressing controversies and divergences in evaluating various forms of clinical utility evidence for guideline development was also gathered and discussed. To assess economic sustainability, given the limited number of economic analyses evaluating the pre-emptive use of large-scale multi-gene panels, systematic literature reviews on economic evaluations of both single- and multi-gene PGx tests were also collected. Regarding patient acceptability, we first conducted a search of surveys and qualitative studies conducted in Europe. However, due to the limited availability of evidence, studies from other relevant geographic contexts were subsequently searched. For organizational barriers, primary studies, policy documents, literature reviews, and expert commentaries addressing feasibility and implementation considerations for pharmacogenetic testing were collected and analyzed. Following the technical team’s preliminary appraisal of the evidence, early findings from the narrative reviews were presented and discussed during the second SC meeting to refine the interpretation of the literature, identify Italy-specific implementation challenges, and flag additional aspects requiring attention. In parallel, the SC was invited to complete a brief written consultation designed to elicit practical considerations and contextual factors that are often underreported in the published literature, and to inform the subsequent discussion and development of recommendations. Responses were discussed within the SC during the third meeting and reflected consensus-oriented input rather than individual-level data. It is important to note that, during the early phase of the study, discussions focused on evaluating a potential “PGx passport” approach (i.e., a portable, patient-linked record of pre-emptive multigene PGx results intended to be accessible at the point of care) as one possible way to deliver pre-emptive multigene results; however, based on the evidence gathered and subsequent discussions within the SC, the focus shifted toward the broader goal of implementing pre-emptive pharmacogenomics, rather than evaluating a single delivery tool. The synthetized literature evidence on the four research areas discussed throughout the paper is reported in Supplementary Tables 1–5. The set of written consultation questions submitted to the SC is also reported in the Supplementary material.

### 2.3 Recommendations

Based on the collected evidence the technical team developed a set of recommendations, which were subsequently validated by the SC to promote a sustainable and evidence-based implementation of pre-emptive PGx testing strategies in Italy.

## 3 Implementational domains

Four key domains were identified as essential for evaluating the implementation of pre-emptive PGx genotyping strategies within the Italian context: clinical utility, economic sustainability, patient acceptability, and organizational feasibility.

### 3.1 Clinical utility

The implementation of a genetic test within a healthcare system requires robust evidence of its clinical utility, defined as the test’s capacity to improve diagnosis, treatment, management, or prevention of disease, resulting in measurable health benefits for patients (20). In the context of pre-emptive PGx, this involves evaluating both how genetic test results should be used to optimize drug therapy and whether ordering a PGx test before initiating treatment is beneficial, ultimately leading to measurable health benefits (9, 14).

A growing body of evidence is continuously gathered and evaluated by leading organizations involved in the development of PGx guidelines. Among the most influential are the Clinical Pharmacogenetics Implementation Consortium (CPIC) in the United States, the Dutch Pharmacogenetics Working Group (DPWG), the French National Network of Pharmacogenetics (RNPGx) and the Spanish Pharmacogenetics and Pharmacogenomics Society (SEFF) in Europe (21–24). These guidelines provide recommendations for prescribing and adjusting medications based on a patient’s genetic profile, focusing on clinical actions once the genotype is known rather than on when or in whom to perform genetic testing (25). The clinical evidence supporting these guidelines primarily stems from studies focused on pharmacokinetics and therapeutic drug monitoring, rather than from intervention studies (25). However, in recent years, these organizations have increasingly advocated for pre-emptive genotyping, recommending that any PGx testing should be incorporated into routine care when clear clinical benefits are evident. The DPWG developed a clinical implementation score in 2018 to guide healthcare professionals in determining when to order PGx tests before or during treatment (26). This score takes into account four criteria: clinical effect, level of evidence, the number of patients needed to genotype, and the pharmacogenetic information provided in the Summary of Product Characteristics (SmPC) approved by the European Medicines Agency (EMA). Based on this score, PGx testing is categorized into three levels: potentially beneficial, beneficial, and essentia (26). To date, the DPWG considers PGx testing essential for 14 pharmacogenes associated with medications that show well-established gene–drug interactions and clear clinical benefits (27). To date, no Italian pharmacogenomic guidelines are available.

Regulatory authorities, such as the EMA and the Agenzia Italiana del Farmaco (AIFA), also play a crucial role in clinical implementation, integrating PGx information into drug labeling (28). Over the years, the inclusion of PGx data in the SmPC or drug labels has increased, driven by regulatory guidelines and policies in drug discovery and development (29). This information is typically added based on pre- or post-marketing studies, or as a result of safety concerns identified after the drug’s approval. A recommendation from a regulatory agency to perform PGx testing serves as a significant driver for clinical implementation. In 2020, the EMA issued a Direct Healthcare Professional Communication highlighting that patients with partial or complete dihydropyrimidine dehydrogenase (DPD) deficiency face increased fluoropyrimidine toxicity and recommending genotyping or phenotyping before treatment (30). This led to a significant rise in DPD testing prior to therapy initiation across several European countries (31). However, discrepancies persist between regulatory agencies and academic guidelines, reflecting differences in scope, evidence thresholds, and methodological approaches. As of today, EMA/AIFA annotations recommend pre-emptive testing for HLA alleles and for germline variants in CYP2C19, CYP2D6, CYP2C9, UGT1A1, and DPYD, to reduce the risk of adverse events and inform dose adjustments (16, 31). These discrepancies reinforce the need for clearer regulatory requirements and for harmonized guidance across professional guidelines and drug labels. This point has been previously emphasized in Europe, where authors have highlighted that consistent implementation depends on alignment between guideline recommendations and regulatory labeling (29).

In recent years, some studies have been conducted to assess the real-world effectiveness of pre-emptive PGx testing. A recently published review by Chenchula et al. gathered evidence from both RCTs and prospective non-randomized studies conducted in Europe and the United States, providing a promising evidence base for the clinical utility of PGx panels in real-world settings (13). Particularly relevant to the Italian context is the PREPARE study, led by the Ubiquitous Pharmacogenomics Consortium (U-PGx), which marked the first time a pharmacogenetics program had been evaluated in real-world European healthcare systems (32). The PREPARE study was a cluster-randomized implementation study, conducted by the ubiquitous pharmacogenomics consortium, assessing the efficacy of a PGx panel of 50 germline variants across 12 genes (*CYP2B6, CYP2C9, CYP2C19, CYP2D6, CYP3A5, DPYD, F5, HLA-B, SLCO1B1, TPMT, UGT1A1, VKORC1*). A total of 6,944 patient (3,342 following a genotype genotype-guided treatment and 3,602 controls) were enrolled across seven European countries (including Italy) to assess the panel’s clinical effectiveness in different healthcare settings (32). Among 1,558 patients with positive results for a reference gene–drug pairs, ADRs occurred in 152 (21.0%) of the genotype-guided group versus 231 (27.7%) in standard care. Overall, ADR incidence was 21.5% (628/2,923) with genotype guidance versus 28.6% (934/3,270) without (32). The evidence provided by PREPARE, together with secondary analyses conducted by the U-PGx consortium and findings from other studies conducted at St. Jude Children’s Research Hospital, Vanderbilt University, and the Mayo Clinic, supports the clinical utility of pre-emptive PGx in practice (32–34).

Clinical utility has also been assessed using quality-adjusted life-years (QALYs) as a summary outcome in cost–utility frameworks (35–40). Within the PREPARE trial utility values were collected prospectively at multiple time points to quantify quality of life and derive QALYs. In a secondary analysis of the Italian PREPARE cohort focused on gastrointestinal cancer patients treated with fluoropyrimidines and/or irinotecan, mean QALYs were higher in the pharmacogenetics-guided arm than in standard care among participants with complete utility data (35). Other studies conducted in Italy and across Europe and the Midde East focused on single actionable gene–drug pairs, support the potential benefit of pre-emptive PGx strategies in terms of QALYs (36–40). However many models rely on published utility weights and disutility values assigned to severe toxicities, which limits the extent to which QALY estimates reflect quality of life under real-world implementation. Given the potential for PGx testing to affect health-related quality of life through both reductions in treatment-related toxicity and psychological responses to testing and treatment decisions, future studies would benefit from collecting standardized health-utility measures and complementary patient-reported outcomes, alongside clinical endpoints.

Despite growing evidence for the clinical utility of PGx testing, significant gaps remain in demonstrating its effectiveness in complex real-world settings. A major challenge is the lack of evidence for multi-gene panels in patients with polypharmacy, since most guidelines focus on single gene–drug pairs in patients not under polytherapy (41). The use of multiple medications can lead to significant alterations in a patient’s drug exposure, due to interactions that modify the pharmacokinetic pathways. Furthermore, the influence of non-genetic factors, such as diet, smoking, alcohol consumption, and clinical factors including co-morbidity themselves, can also affect drug metabolism (41). For instance, various organizations and companies have developed decision support systems (CDS) to evaluate phenoconversion (i.e., the phenomenon where an individual’s drug metabolism phenotype differs from what would be predicted based on genotype due to non-genetic factors) in polytherapy and to integrate genetic and non-genetic data but their efficacy and feasibility in clinical practice are still under investigation (42, 43).

### 3.2 Economic sustainability

A meta-analysis of observation studies published in 2016 estimated that the average cost of a single ADR for the healthcare system was €2,454.44 (95% CI: 1,164.24–3,744.64) (44). From these data the authors estimated that in Tuscany, the total expenditure for ADRs would have been €3.4 million per million inhabitants (95% CI: 1.7–5.1 million) in 2016, with estimated potential savings of €1.5 million per million inhabitants (95% CI: 0.78–2.3 million) for preventable ADRs, defined as those avoidable through appropriate measures during prescribing, dispensing, administration, or monitoring (44). As genetic testing costs continue to decline driven by advances in next-generation sequencing, PGx testing emerges as a promising strategy to reduce ADR-related expenses and improve patient safety and treatment outcomes. However, conducting robust economic evaluations presents several challenges related to a lack of clinical efficacy data from RCTs, limited real-world ADR incidence data, and uncertainty in genetic variant prevalence across European countries (45–48).

Despite this, numerous economic evaluations have been conducted on various PGx panels (48–52). A systematic review encompassing 47 economic evaluations found strong support for the cost-effectiveness of specific pharmacogenomic tests, including HLA-B*57:01 for abacavir hypersensitivity and *CYP2C19* genotyping for clopidogrel therapy (48). A second review focusing on PGx-guided therapies included 80 economic evaluations and reported that most studies found PGx-guided treatment to be cost-effective or even cost-saving. However, results varied depending on several factors, including biomarker prevalence, test cost, willingness-to-pay thresholds, ADR incidence, and therapeutic response rates (39). Similarly, a recent review of 108 studies covering 39 drugs found that 71% of the PGx tests evaluated were cost-effective or cost-saving, confirming the economic value of testing for clopidogrel, warfarin and several antidepressants, while data on pre-emptive and multi-gene panel testing remain limited (40). Collectively, these findings underscore the potential economic benefits of PGx testing, while also highlighting the need for more comprehensive and context-specific evaluations, particularly for broader panel-based approaches. Cost utility analyses of pre-emptive multigene panels in different European settings have been conducted within the U-PGx project, based on the results of the multicenter PREPARE trial (35, 53–55). The analysis conducted in Italy on colorectal cancer patients showed that PGx-guided care reduced overall treatment costs per patient (€380 vs. €565 in the control arm), while increasing mean survival by 0.25 life-years (35).

### 3.3 Patient acceptability

Patient acceptance of PGx testing is essential for its successful integration into healthcare systems, as patient willingness directly impacts the successful implementation of these tests. However, studies on acceptability of pharmacogenetic testing in the Italian and European context are limited.

A large UK survey found that predicted uptake of pharmacogenetic testing services could range from 51% to over 99%, depending on how the service was designed, with preferences for noninvasive testing, data access, and regional data sharing (56). Studies conducted in United States and in Canada confirm that most patients express favorable attitudes toward pharmacogenetic testing when adequately informed and are comfortable with clinicians using these results to guide care (57–60).

However, several studies have shown that PGx testing may face acceptance challenges related to limited patient awareness and concerns about privacy or misuse of genetic data. A recent scoping review of implementation in primary care identified low patient awareness, as well as ethical and legal concerns about pharmacogenomic data, as major challenges (61). A qualitative systematic review and survey-based studies further confirm that patients often lack knowledge about pharmacogenetic testing, express concerns about privacy, insurance-related discrimination, and perceived negative implications of test results, and may be skeptical about the utility of testing if not well informed (62). These factors directly influence willingness to participate in testing and are particularly pronounced in underserved or minority populations (63). Pilot implementation studies have also shown that even when testing is offered at no cost, participation rates can be suboptimal due to lack of understanding, privacy concerns, and a desire for more personalized education and counseling about test results (56–64). Collectively, these findings underscore that addressing knowledge gaps, privacy concerns, and trust issues is essential to improve acceptability and successively implement pre-emptive PGx.

### 3.4 Organizational feasibility

Our literature review and stakeholder consultations revealed several organizational barriers, including the lack of reimbursement strategies, disparities in the access to testing services, inadequate laboratory infrastructure, and gaps in knowledge and training among healthcare professionals (33, 58, 65–70).

In Italy, the reimbursement for genetic testing within the national health service is governed by the essential levels of care (*livelli essenziali di assistenza*, LEA) framework, which outlines the services and treatments available to all citizens, either free of charge or with a small fee (range 1–36€), as a cost-sharing mechanism. However, the recent updates to the LEA have excluded several PGx tests that were previously approved by the EMA, including DPYD (17). This exclusion is likely to exacerbate disparities at regional level, with some tests being available under different nomenclatures or limited to research institutions. As a result, in Italy the implementation of PGx testing remains inconsistent, with the lack of a clear and standardized reimbursement structure hindering the widespread adoption of these technologies across the country.

Infrastructural challenges also remain a critical issue, with disparities in the availability of next generation sequencing platforms for PGx testing across different regions. Data from the *Agenzia nazionale per i servizi sanitari regionali* show a concentration of testing facilities in certain areas of Italy, while other regions, particularly the South and Islands, have limited access to these services (65). The survey revealed Lombardy had the highest number of facilities (*n* = 28), followed by Sicily (*n* = 15) and Lazio (*n* = 13). Overall, there were 67 facilities providing these services in the North, 24 in the Center, 23 in the South, and 17 in the Islands, of which only 2 are in Sardinia (*n* = 44) (65).

Another critical challenge lies in the lack of widespread, robust infrastructures for the storage, management, analysis, interpretation, and sharing of pharmacogenetic data (11, 12, 33, 66). This gap is a major obstacle to integrating PGx information into clinical workflows and electronic health records, ultimately limiting the ability to deliver actionable PGx recommendations at the point of care. Additionally, the absence of standardized and interoperable systems for sharing PGx results across institutions further exacerbates these challenges, leading to fragmented implementation and hindering scalability. Experience from the U-PGx project reveals also challenges in the development and deployment of CDS tools for PGx primarily driven by institutional challenges, and complexities involved in building and maintaining reference data resources, such as genotype–phenotype mappings (33). Even after the implementation of CDS tools, issues such as harmonizing PGx information and ensuring compliance with regulatory frameworks persisted.

Another key concern is the lack of PGx knowledge among healthcare professionals (11, 12, 58, 66–69). A survey conducted in South Italy revealed that although a large proportion of residents (93%) and physicians (79%) acknowledged the utility of PGx tests, only a small fraction felt confident in selecting and interpreting these tests based on their training (66). This educational gap represents a broader, transnational issue, as documented not only in Italy but across Europe, as reported by several studies, including those conducted by the U-PGx consortium (58, 67–69). This highlights a need for improved education and targeted training, particularly to address the varying needs of different healthcare professionals. Education and training programs have been shown to be effective in increasing confidence and promoting PGx testing (70). Notably, primary care physicians tend to find PGx testing more useful than specialists, and educational interventions have led to significant improvements in their ability to order PGx tests.

## 4 Recommendation for a sustainable implementation

Over the last decade, several healthcare systems have begun to move from isolated, single gene–drug testing toward more systematic approaches to pre-emptive PGx, supported by national or health-system initiatives and, in some settings, established guideline infrastructures. In the Netherlands, long-standing pharmacogenetic guidance has provided a reference framework for translating gene–drug evidence into prescribing recommendations, while ongoing implementation work continues to address operational barriers (71, 72). In the UK, discussions within the National Health Service have increasingly emphasized the potential role of panel-based pre-emptive testing and the need for service models that enable routine access to results at the point of care (73, 74). In the United States, multiple health systems have operationalized pre-emptive PGx through EHR-integrated programs, highlighting the importance of clinical decision support and interoperable data pathways for translating test results into actionable prescribing (34, 75, 76). Taken together, these experiences suggest that achieving routine, sustainable pre-emptive PGx requires coordinated action across governance, evidence generation, professional training, laboratory capacity, and information systems. In line with these international lessons and the other evidence reviewed in this study, we identified a set of strategic actions to support the effective implementation of pre-emptive PGx testing in Italy (Table 1). These actions are grouped into six categories: (1) regulatory actions, (2) research investments, (3) professional training and regulatory frameworks for result interpretation, (4) public awareness and informed consent, (5) laboratory infrastructure, and (6) information technology (IT) systems and data protection.

**Table 1:** Recommended actions and key stakeholders for the effective implementation of pre-emptive PGx testing in Italy.

Area	Recommended actions	Stakeholders
Regulatory actions	Integrate PGx recommendations into national clinical guidelines in key specialties.	National scientific societies Ministry of Health AIFA Academic and research institutions ISS Professional medical associations
	Develop and implement reimbursement policies for pharmacogenetic testing as part of the National Essential Level of Care (LEA).	Ministry of Health National and Regional Health Authorities AIFA AGENAS ISS Scientific societies and patient advocacy groups
	Implement a national regulatory framework to standardize PGx tests results and ensure interpretation is performed by trained professionals.	Ministry of Health National and Regional Health Authorities ISS Scientific societies Professional medical associations
	Revise SmPC language to improve clarity and strengthen PGx testing recommendations for relevant gene-drug pairs.	AIFA Ministry of Health EMA National scientific societies Pharmaceutical Companies Association Clinical pharmacologists and pharmacogenomics experts/Geneticists
Research investments	Fund implementation studies on the clinical utility and cost-effectiveness of multi-gene PGx panels and decision support tools.	Ministry of University and Research Ministry of Health Academic and research institutions Public and private research funding bodies Pharmaceutical and medical devices companies Scientific societies Professional medical associations
	Support international research consortia to generate generalizable evidence on the real world PGx implementation.	Ministry of University and Research International research consortia Academic and research institutions Public and private research funding bodies Pharmaceutical and medical devices companies Scientific societies Professional medical associations
Professional training and regulatory frameworks for result interpretation	Develop training programs to improve clinicians’ ability to interpret and use PGx results in clinical care.	Ministry of Health Ministry of University and Research AIFA National scientific societies Professional medical associations Regional health authorities
	Create frameworks and structured re-training initiatives to ensure PGx result interpretation evolves with updated guidelines and tools.	Ministry of Health Ministry of University and Research Professional medical associations Regional health authorities
Public awareness and informed consent	Promote public education campaigns to build understanding and trust in PGx testing and pre-emptive multi-gene PGx panels.	Ministry of Health ISS Regional health authorities Patient advocacy groups and associations Academic and research institutions Public communication agencies General practitioners associations
	Develop standardized informed consent procedures that clearly explain PGx testing implications.	Ministry of Health ISS Regional health authorities Patient advocacy groups and associations National Bioethics Committee Legal and ethics experts
Laboratory infrastructure	Establish a national accreditation framework to ensure quality and standardization in PGx testing laboratories.	Ministry of Health ISS Regional Health Authorities Clinical laboratories and diagnostic centers Scientific societies Quality assurance and regulatory experts Universities and research institutions Private companies developing diagnostic kits
	Invest in laboratory infrastructure to expand equitable access to PGx testing across all regions, with a focus on underserved areas.	Ministry of Health ISS Regional Health Authorities Ministry of Economy and Finance Public hospital networks Clinical laboratories and diagnostic centers Scientific societies EU funding programs and national recovery/resilience initiatives
IT systems and data protection	Define and implement national standards for PGx data integration and interoperability across IT systems, including the FSE.	Ministry of Health ISS Agency for Digital Italy Regional Health Authorities Health IT providers and vendors Hospitals networks Developers of clinical decision support systems
	Establish legal and ethical frameworks for PGx data use, aligned with GDPR and privacy protections.	Ministry of Health ISS Garante per la Protezione dei Dati Personali Ministry of Justice National Bioethics Committee Legal experts and ethics experts Scientific societies Patient advocacy groups

ISS, Istituto Superiore di Sanità (National Institute of Health); AIFA, Agenzia Italiana del Farmaco (Italian MedicinesAgency); EMA, European Medicicines Agency.

### 4.1 Regulatory actions

Effective integration of PGx testing in Italy requires national clinical guidelines aligned with international recommendations and standardized reimbursement frameworks. Guidelines in oncology, cardiology and psychiatry must be updated to include PGx tests with strong evidence recommended by international organizations such as the DPWG, ensuring consistent, evidence-based testing nationwide. Scientific societies in the field of pharmacology (SIF) should work with medical professionals in different fields to create PGx guidelines that may be integrated into already existing formal clinical pathways for each specific disease setting.

In addition to updating clinical guidelines, SmPCs should be revised to include clearer and more easily actionable recommendations regarding PGx testing. A recent analysis comparing PGx information in AIFA-approved drug labels with those of other regulatory bodies revealed significant gaps in clarity (30). This highlights the need for standardized, unambiguous labeling to support clinicians implementing PGx testing into routine practice. Moreover, clear and standardized reimbursement pathways are essential for the widespread adoption of PGx testing. A key step is ensuring that pharmacogenetic tests are included in the LEA according to existing PGx recommendations, making them accessible, prescribable for the most appropriate actionable gene–drug interactions, and covered by the Italian national health service. This inclusion would mitigate regional disparities, streamline PGx integration into routine care while driving an appropriate application of PGx in the diagnostic routine.

### 4.2 Research investments

While existing evidence is sufficient to support the recommendation of pre-treatment testing for certain gene-drug pairs, as indicated by international PGx guidelines, more comprehensive research is needed to establish the most effective implementation strategies and to conduct the necessary economic evaluations to assess the long-term implications of pre-emptive genotyping strategies. Priority should be given to research assessing the clinical effectiveness of multi-gene PGx panels and CDS across various clinical contexts. Such evidence is essential for identifying appropriate clinical pathways for PGx testing and understanding its feasibility and benefits in real-world settings. Moreover, the findings from these studies will provide the necessary foundation for conducting economic evaluations of PGx testing. These evaluations are crucial to compare the costs and benefits of implementing multi-gene panels in various healthcare contexts, while also assessing the economic and clinical impact that the re-usability of the PGx data may have in the long term.

### 4.3 Professional training and regulatory frameworks for result interpretation

Despite the growing awareness of the clinical utility of PGx, many healthcare providers still lack the necessary knowledge and confidence to effectively incorporate PGx information into their clinical decision-making. To ensure the accurate and reliable application of PGx data, it is essential that test results be interpreted by healthcare professionals with specific training in PGx. Such expertise is critical to minimizing misinterpretation risks and preventing potential harm to patients. However, the full potential of pre-emptive multi-gene panels lies not only in their initial application but also in the capacity to utilize genetic results across the patient care continuum. This requires the development of targeted educational programs that improve clinician understanding of PGx testing, including how to interpret results and integrate them into treatment decisions. Recommendations for using and interpreting PGx results should evolve over time, aligned with advancements in professional training and the ongoing development and validation of decision support systems.

### 4.4 Public awareness and informed consent

Ensuring that patients fully understand the potential benefits and risks of PGx testing, as well as the impact on their treatment, is essential for increasing acceptance and participation in these programs. Public awareness campaigns and educational initiatives targeting both patients and healthcare professionals are needed to foster trust in PGx testing and address concerns related to privacy, discrimination, and the potential psychological impact of genetic results. Moreover, the process of informed consent should be carefully structured to ensure that patients are fully aware of the implications of undergoing PGx testing, including potential privacy concerns and the consequences of finding genetic variants that may not be directly relevant to their immediate care.

### 4.5 Laboratory infrastructure and PGx report development

Given the complexity and technical demands of PGx, it is essential that laboratories adhere to rigorous quality standards to ensure the accuracy, reliability, and reproducibility of test results. Establishing a nationwide accreditation framework for PGx laboratories would promote uniformity across Italy, ensuring that genetic tests are conducted following standardized protocols and that results are consistently interpreted. This will not only enhance the credibility of PGx testing but also reduce potential errors associated with inconsistent laboratory practices. Currently a high heterogeneity exists in the type of laboratories and professionals that deliver the PGx test, leading to sometimes inconsistent reporting and interpretation of the results. A minimum set of information to be included in the PGx report should be defined, including a reference to the PGx guidelines on which the analysis is based. In addition, clinical pharmacology counseling for the interpretation of the results should always be recommended, potentially considering other relevant information such as pharmacological interactions and therapeutic drug monitoring. Furthermore, the distribution of these testing services needs to be expanded. It is crucial to invest in the infrastructure of laboratories in regions with limited access to ensure that PGx services are available nationwide. Important in this context is also the role of private companies in creating commercial diagnostic kits under EU IVDR, providing standardized molecular diagnostics that are easy to use in peripheral centers.

### 4.6 IT systems and data protection

A major challenge is ensuring interoperability of healthcare IT systems across regions, particularly to enable seamless, standardized sharing of PGx data. The absence of uniform data formats and platforms for exchanging PGx information significantly impedes the widespread adoption and integration of these tests into clinical practice. Moreover, the currently used system for PGx data reporting and storage is essentially based on reports stored in an unstructured format, not questionable by the users and not providing interruptive alerts in case of prescription errors on the PGx basis. To address this, it is crucial to establish national standards for PGx data integration in electronic clinical records, ensuring compatibility between systems used in laboratories, clinics, and healthcare institutions.

Furthermore, alongside the technical challenges, there are important legal and ethical considerations surrounding the use of PGx data. Privacy concerns are paramount, particularly regarding the protection of genetic information. Ensuring confidentiality and building patient trust require stringent adherence to data protection regulations, such as the GDPR in Europe. It is essential to create robust legal frameworks for the collection, storage, and sharing of PGx data, protecting patient rights while ensuring that this sensitive information is used in a responsible and ethically manner.

## 5 Conclusion

Pre-emptive PGx testing holds considerable potential to improve patient’s outcomes, medication safety, and healthcare sustainability in Italy. Nonetheless, several barriers still hinder its widespread adoption. Translating the strategic priorities identified in this study into actionable policies and structured clinical practices is essential to support the integration of PGx into the Italian healthcare system in a coordinated and equitable manner. Since many of these barriers are also encountered in other European countries, collaborative research efforts and structured exchanges of best practices could further enhance implementation across Europe.
