# Supplementary material for: Pre-emptive pharmacogenetic testing in Italy: a review of evidence and multidisciplinary consensus on key priorities for implementation
Source: Front Public Health. 2026 Mar 12;14:1763975. doi: 10.3389/fpubh.2026.1763975 (PMC13018106; doi:10.3389/fpubh.2026.1763975)
Supplement: Supplementary file 1 [file Table_1.DOCX]

Supplementary Material

**Implementing Pre-emptive Pharmacogenetic Testing in Italy: A Review of Evidence and Multidisciplinary Consensus on Key Priorities**

[Supplementary Table 1. Collected evidence on the clinical utility of pre-emptive PGx 2](#_Toc201680211)

[Supplementary Table 2. Collected evidence on economic sustainability of pre-emptive PGx 5](#_Toc201680212)

[Supplementary Table 3. Collected evidence on patients acceptability of pre-emptive PGx 9](#_Toc201680213)

[Supplementary Table 4. Collected evidence on organizational feasibility of pre-emptive PGx 11](#_Toc201680214)

[Steering Committee Consultation Questionnaire 14](#_Toc201680215)

[References 15](#_Toc201680216)

Supplementary Table 1. Collected evidence on the clinical utility of pre-emptive PGx

| **Title** | **Authors (Year), Country** | **Type of study** | **Key findings** |
| --- | --- | --- | --- |
| A 12-gene pharmacogenetic panel to prevent adverse drug reactions: an open-label, multicentre, controlled, cluster-randomised crossover implementation study. [1] | Swen et al. (2023), various countries | Cluster-randomised, controlled trial. | Use of a 12-gene pharmacogenetic panel reduced clinically relevant adverse drug reactions by ~30%. Among patients with actionable variants, ADRs occurred in 21.0% (intervention) vs. 27.7% (control), OR 0.70. In the full population, ADRs were 21.5% vs. 29.0%, OR 0.70. Supports large-scale implementation of pre-emptive pharmacogenetic testing. |
| Clinical Impact of Pharmacogenetic Profiling with a Clinical Decision Support Tool in Polypharmacy Home Health Patients. [2] | Elliott et al. (2017), USA | Randomized controlled trial | Among the 110 polypharmacy patients enrolled, patients who underwent pharmacogenetic testing had significantly fewer adverse clinical events compared to untested patients after 60 days. The treated group experienced 52% fewer re-hospitalizations, 42% fewer emergency department visits and a 48% reduction in the combined rate of re-hospitalizations and ED visits. |
| The effect of pharmacogenetic profiling with a clinical decision support tool on healthcare resource utilization and estimated costs in the elderly exposed to polypharmacy. [3] | Brixner et al. (2015), USA | Prospective observational study | In this propensity score–matched cohort (205 tested vs. 820 untested patients), pharmacogenetic testing was associated with a 39% reduction in hospitalization rates and a 71% reduction in emergency department (ED) visit rates. In contrast, the rate of outpatient visits was 96% higher in the tested group compared to the untested group. |
| Pharmacogenetic-Guided Psychiatric Intervention Associated With Increased Adherence and Cost Savings. [4] | Fagerness et al. (2014), USA | Retrospective observational study | 111 tested patients (cases) were compared with 222 matched untested controls. At baseline, adherence was significantly lower in tested patients (85.2%) than in controls (92.8%). However, over the follow-up period, patients who received the pharmacogenetic test showed a 6.3% increase in medication adherence compared to only 0.3% in controls, resulting in a statistically significant net improvement of 6.0%. |
| Clinically actionable genotypes among 10,000 patients with preemptive pharmacogenomic testing. [5] | Van Driest et al. (2014), USA | Descriptive implementation study | Among the 9,589 patients with complete genotype data for five drug-gene interactions (DGIs), 91% had at least one actionable variant, those with a known impact on drug response and linked to clinical decision support in the electronic medical record. The rate was even higher among African American patients (96%). 42% of patients with actionable variants had already been exposed to the relevant medication, emphasizing the clinical relevance of preemptive testing. When compared to a reactive, prescription-triggered testing strategy, the preemptive panel approach required significantly fewer tests (9,589 vs. 14,656) while ensuring that genetic data were available at the point of care. |
| PG4KDS: A Model for the Clinical Implementation of Pre-emptive Pharmacogenetics [6]. | Hoffman et al. (2014), USA | Descriptive implementation study | 1,559 pediatric patients were preemptively genotyped and results integrated into the EHR with clinical decision support linked to 12 high-risk drugs. Among 1,016 patients with results, 78% had at least one actionable genotype. |
| Preemptive Genotyping for Personalized Medicine: Design of the Right Drug, Right Dose, Right Time – Using Genomic Data to Individualize Treatment Protocol. [7] | Bielinski et al. (2014), USA | Descriptive implementation study | Patients at high risk of statin were identified performing preemptive genotyping of 84 pharmacogenes and CYP2D6. Among 2000 invited Biobank participants, 50% provided blood samples. The CDS alerts were created for several drug-gene pairs (e.g., abacavir/HLA-B*5701, thiopurines/TPMT), and integration into EMR allowed real-time therapeutic guidance. The project emphasized the feasibility of preemptive PGx testing, demonstrating potential for more efficient, safe, and personalized prescribing, while also identifying barriers like provider education and system integration. |
| The CLIPMERGE PGx Program: Clinical Implementation of Personalized Medicine Through Electronic Health Records and Genomics–Pharmacogenomic. [8] | Gottesman et al. (2013), USA | Descriptive implementation study | Preemptive genotyping for key pharmacogenomic variants (e.g., CYP2C19, CYP2C9, VKORC1, SLCO1B1, CYP2D6) was integrated into the EHR via a custom-built CDS system. Real-time, point-of-care alerts were generated when genotype-drug interactions were detected, including actionable recommendations and dosing suggestions. Providers receiving CDS were trained and surveyed. |
| Concordance of DMET Plus Genotyping Results With Those of Orthogonal Genotyping Methods. [9] | Fernandez et al. (2012), USA | Validation study | The Affymetrix DMET Plus array, a high-throughput pharmacogenetic platform that interrogates 1,931 variants across 225 ADME-related genes, was assessed by comparing its genotyping results with those from several orthogonal methods in 220 pediatric patients. After applying quality control filters, 1,692 variants were retained, and 259 were cross-validated using other genotyping techniques. Among nearly 20,000 SNP–patient sample pairs, the overall concordance rate was exceptionally high at 99.9%, and for eight clinically actionable genes (e.g., *TPMT*, *CYP2D6*, *VKORC1*), the concordance reached 99.96%. Only six SNPs fell below the 98% concordance threshold, mainly due to discrepancies with the SNPstream method, and evidence in several cases supported the DMET result. |
| Implementing Personalized Medicine: Development of a cost-Effective customized Pharmacogenetics Genotyping Array. [10] | Johnson et al. (2012), USA | Descriptive implementation study | A pharmacogenetics genotyping array twas designed based on curated clinical annotations from PharmGKB and includes 256 SNPs across 120 genes relevant to drug metabolism, transport, and response. Using the QuantStudio 12K Flex OpenArray platform, the array offers a fast (5-hour) turnaround, low per-sample cost (~$42), and flexibility to update content as new evidence emerges. Initially focused on guiding clopidogrel therapy through CYP2C19 genotyping, the platform supports broader integration of pharmacogenetic data into electronic medical records, enabling its use when needed. |
| Assessment of a pharmacogenomic marker panel in a polypharmacy population identified from electronic medical records. [11] | Oetjens et al. (2013), USA | Cross-sectional study | The performance of the Illumina ADME Core Panel in genotyping pharmacogenetic variants was assessed using data from 326 polypharmacy patients of European descent, who were selected from Vanderbilt’s BioVU biobank. High genotyping quality was demonstrated, particularly for diallelic variants, although reduced performance was observed for certain copy number variants (CNVs) and markers in genes such as *CYP2D6*. Many of the variants targeted by the panel were found to be rare or monomorphic, thereby highlighting limitations when fixed-content panels are applied to smaller samples. Good concordance with the HumanOmni1-Quad array was shown, but it was also revealed that numerous pharmacogenomic variants were not captured or adequately tagged by genome-wide association study (GWAS) arrays. Allele frequencies observed in the sample were found to be largely consistent with those of European reference populations. |

# **Supplementary Table 2. Collected evidence on economic sustainability of pre-emptive PGx**

| **Title** | **Authors (Year), Country** | **Type of study** | **Key findings** |
| --- | --- | --- | --- |
| Potential Direct Costs of Adverse Drug Events and Possible Cost Savings Achievable by their Prevention in Tuscany, Italy: A Model-Based Analysis. [12] | Convertino et al. (2019), Italy | Model base analysis | The study estimated the mean cost per adverse drug event (ADE) at €2471.46 using Monte Carlo simulations and found that 45% of ADEs were preventable. By applying these estimates to pharmacovigilance data from Tuscany (2016), a regional expenditure of approximately €3.4 million per million inhabitants was projected due to ADEs. Notably, over €1.5 million per million inhabitants could potentially be saved through the prevention of avoidable ADEs. |
| Cost-Effectiveness of Panel Tests for Multiple Pharmacogenes Associated With Adverse Drug Reactions: An Evaluation Framework. [13] | Plumpton et al. (2019), UK | Cost-effective analysis | The study found that, when all actionable genetic variants were considered irrespective of individual cost-effectiveness, use of the HLA gene panel resulted in cost savings of £378 (US$491) and a gain of 0.0069 quality-adjusted life-years (QALYs). Stratified analyses showed the panel to be cost-effective for patients eligible to receive abacavir (HLA-B57:01), carbamazepine (HLA-A31:01), and clozapine (HLA-B (158T) and HLA-DQB1 (126Q), but not for carbamazepine (HLA-B15:02) or allopurinol (HLA-B58:01). |
| Economic evaluation of genomic/genetic tests: a review and future directions. [14] | Bouttell et al. (2022) | Review |  |
| Pharmacogenetic testing for adverse drug reaction prevention: systematic review of economic evaluations and the appraisal of quality matters for clinical practice and implementation. [15] | Turongkaravee et al. (2021) | Systematic review | The review found that pharmacogenetic testing is frequently cost-effective or cost-saving for the prevention of adverse drug reactions, particularly when allele frequencies are high and effective therapeutic alternatives are available. Cost-effectiveness was often demonstrated for drugs such as warfarin (CYP2C9/VKORC1), clopidogrel (CYP2C19), abacavir (HLA-B57:01), efavirenz (CYP2B6), azathioprine (TPMT), carbamazepine (HLA-B15:02, HLA-A*31:01), irinotecan (UGT1A1), and fluoropyrimidines (DPYD). Cost-effectiveness was found to vary depending on the country, population, and available drug alternatives, underscoring the need for context-specific assessments. |
| A Systematic Review of Economic Evaluations of Pharmacogenetic Testing for Prevention of Adverse Drug Reactions. [16] | Plumpton et al (2016), UK | Systematic review | The study identified 47 economic evaluations of pharmacogenetic testing. Evidence supported the cost-effectiveness of testing for HLA-B*57:01 (prior to abacavir), HLA-B*15:02 and HLA-A*31:01 (prior to carbamazepine), HLA-B*58:01 (prior to allopurinol), and CYP2C19 (prior to clopidogrel). In contrast, economic evidence was inconclusive for TPMT (before 6-mercaptopurine, azathioprine, and cisplatin), CYP2C9 and VKORC1 (for genotype-guided dosing of coumarins), MTHFR (prior to methotrexate), and factor V Leiden (prior to oral contraceptives). Testing for A1555G prior to aminoglycoside therapy was found not to be cost-effective. |
| Economic Evaluations of Pharmacogenetic and Pharmacogenomic Screening Tests: A Systematic Review. Second Update of the Literature. [17] | Berm et al. (2016) | Systematic review | The review analyzed 80 economic evaluations of pharmacogenetic (PGx) testing. Most studies concluded that PGx tests were cost-effective or cost-saving; however, few assessed the intrinsic value of the test independently of specific drug-treatment pairings. Instead, cost-effectiveness was often tied to drug prices, reflecting a tendency to bundle PGx testing with particular therapies. The review also observed an increase in industry-funded studies since 2010, all of which reported favorable outcomes, raising concerns about potential bias, particularly given the limited discussion of analytical validity and direction of bias. PGx test costs ranged widely (US$10 to $575). |
| Cost Effectiveness of Pharmacogenetic Testing for Drugs with Clinical Pharmacogenetics Implementation Consortium (CPIC) Guidelines: A Systematic Review. [18] | Morris et al. (2022), USA | Systematic review | The review identified 108 studies evaluating PGx testing across 39 drugs. Of these, 71% (n = 77) found PGx testing to be cost-effective (n = 48) or cost-saving (n = 29), while 20% (n = 21) reported it was not cost-effective, and 9% (n = 10) yielded uncertain results. Clopidogrel was the most frequently studied drug (n = 23), with 22 studies reporting favorable economic outcomes, followed by warfarin (n = 16), with 7 studies showing cost-effectiveness or cost-saving results. Among 26 studies assessing HLA testing for abacavir, allopurinol, or carbamazepine/phenytoin, 15 supported its economic value. Likewise, 9 of 11 studies on antidepressants reported cost-effectiveness or cost-saving outcomes. The median Quality of Health Economic Studies (QHES) score was high (median 91; range 48–100), indicating overall good methodological quality. |
| Cost effectiveness of pharmacogenomics: a critical and systematic review. [19] | Wong et al. (2010) | Systematic review | The review evaluated 34 economic evaluations of PGx interventions, with 91% of studies published after 2004 and a growing focus on cost-utility analyses using quality-adjusted life-years (QALYs), especially in the final three years of the review period. Thromboembolic diseases and cancer were the most frequently studied conditions, while TPMT and CYP2C9 were the most assessed biomarkers. Study quality was generally moderate to high, with an average Quality of Health Economic Studies (QHES) score of 77 out of 100. Although 63% of biomarkers showed clinical validity, only HER-2 and HLA-B57:01 had strong evidence of clinical utility. The cost-effectiveness of PGx testing varied widely: some interventions, such as HLA-B57:01 screening in HIV and Oncotype DX in breast cancer, were found to be highly cost-effective or cost-saving, whereas others, like genotype-guided warfarin dosing, produced mixed results depending on model assumptions. |
| Cost-Effectiveness of Pharmacogenomic and Pharmacogenetic Test-Guided Personalized Therapies: A Systematic Review of the Approved Active Substances for Personalized Medicine in Germany. [20] | Plöthner et al. (2016), Germany | Systematic review | The review identified 27 economic evaluations examining the cost-effectiveness of PGx -guided therapies. In most cases, PT-guided administration of active ingredients was found to be either cost-effective or cost-saving. However, no clear consensus emerged regarding the cost-effectiveness of test-guided therapies when applied independently of the therapeutic indication. Most studies focused on targeted therapies in oncology. The cost-effectiveness of PT-guided approaches was shown to depend on multiple factors, including biomarker prevalence, test costs, threshold values, the prevalence of adverse drug reactions (ADRs), and treatment response rates. Considerable variability in cost-effectiveness was reported both across different clinical indications and within the same indication. |
| Cost-utility analysis of pharmacogenomics-guided tacrolimus treatment of Slovenian patients undergoing kidney transplantation in the U-PGx PREPARE study. [21] | Fragoulakis et al (2025), various countries | Cost-utility analysis | The study found that PGx-guided tacrolimus therapy in kidney transplant patients, tailored to the patient’s CYP3A5 genotype, led to improved quality-adjusted life-years (QALYs) (0.956 vs. 0.862) and a 43% greater improvement in quality of life. While total healthcare costs were similar between groups (€4689 vs. €4650), the PGx-guided approach eliminated severe adverse drug events (ADEs), including deaths, and reduced grade 2 ADEs. The incremental cost-effectiveness ratio (ICER) favored the PGx strategy, indicating it to be a cost-effective and potentially dominant option. |
| Cost-utility analysis of pharmacogenomics-guided tacrolimus treatment in Austrian kidney transplant recipients participating in the U-PGx PREPARE study. [22] | Fragoulakis et al. (2024), various countries | Cost-utility analysis | The study found that PGx-guided tacrolimus therapy in Austrian kidney transplant recipients was both clinically beneficial and cost-effective compared to standard treatment. Patients receiving PGx-guided therapy experienced fewer and less severe adverse drug events (ADEs), required fewer hospitalization days, and achieved slightly higher quality-adjusted life-years (QALYs). From an economic perspective, the PGx-guided group incurred €3902 less in healthcare costs per patient, with most savings attributed to reduced hospitalization. The incremental cost-effectiveness ratio (ICER) favored the PGx approach, indicating it was not only more effective but also less costly. |
| Cost-utility analysis and cross-country comparison of pharmacogenomics-guided treatment in colorectal cancer patients participating in the U-PGx PREPARE study. [23] | Fragoulakis et al. (2023), various countries | Cost-utility analysis | The study estimated total costs of €380 (∼US$416; 95% CI: 195–596) for the PGx-guided arm, compared to €565 (∼US$655; 95% CI: 340–724) in the control arm. Mean survival was higher in the PGx-guided group (1.58 ± 0.25 life-years) versus the control group (1.50 ± 0.26), with a statistically significant difference (Log Rank test, χ² = 4.219; p = 0.04). No significant differences were observed in quality-adjusted life-years (QALYs). The incremental cost-effectiveness ratio (ICER) was €13,418 (∼US$14,695) per QALY. The cost-effectiveness acceptability curve showed that at a willingness-to-pay threshold below €5000, the probability of PGx being cost-effective exceeded 70%. |
| Economic evaluation of pharmacogenomic-guided antiplatelet treatment in Spanish patients suffering from acute coronary syndrome participating in the U-PGx PREPARE study. [24] | Koufaki et al. (2023), various countries | Economic evaluation | The study evaluated the cost-effectiveness of pharmacogenomic (PGx)-guided clopidogrel therapy compared to standard care in 243 Spanish patients with acute coronary syndrome (ACS). PGx-guided treatment resulted in fewer hospital admissions, emergency visits, and adverse drug reactions, alongside slight improvements in quality-adjusted life-years (QALYs) and life expectancy. The total mean cost per patient in the PGx group was nearly half that of the control group (€883 vs. €1,755), despite including the cost of genetic testing. |

# **Supplementary Table 3. Collected evidence on patients acceptability of pre-emptive PGx**

| **Title** | **Authors (Year), Country** | **Type of study** | **Key findings** |
| --- | --- | --- | --- |
| Public preferences for pharmacogenetic testing in the NHS: Embedding a discrete choice experiment within service design to better meet user needs. [25] | McDermott et al. (2024), UK | Discrete choice experiment | Respondents showed strong interest in pharmacogenetic testing when offered in primary care, with predicted uptake ranging from 51% to over 99% depending on test features. Preferences were consistently in favour of noninvasive sampling (cheek swab), patient access to results, and reuse of data for future prescriptions. Regional data sharing was slightly preferred over national models. |
| Lessons from clinical implementation of a preemptive pharmacogenetic panel as part of a testing pilot program with an employer-sponsored medical plan. [26] | Norris et al. (2023), USA | Implementation study | Most participants viewed the pharmacogenetic testing process positively, though only a quarter believed the results would influence their care. Despite 89% of tested individuals having at least one actionable genotype, many expressed difficulty understanding their results, and over 70% said they would have preferred a consultation with a specialist. Patient engagement was limited by a general lack of familiarity with pharmacogenetics, uncertainty about benefits, and concerns about data use. |
| Healthcare provider and patient perspectives on the implementation of pharmacogenetic-guided treatment in routine clinical practice. [27] | Kaur et al.,  (2018), Canada | Survey | 52% of patient respondents were aware of PGx testing, with a significant association between awareness and positive opinions toward PGx. Both healthcare providers and patients recognized the value of Point of Care (PoC) PGx testing devices, with 98% of healthcare providers and 71% of patients believing PoC devices would improve the accessibility and implementation of PGx testing. |
| Pharmacogenetic Testing in Primary Care Practice: Opinions of Physicians, Pharmacists and Patients. [28] | Frigon et al. (2019), Canada | Qualitative study | Most patients expressed interest in PGx testing, highlighting its potential to reduce adverse effects and improve treatment. However, many preferred physicians over pharmacists to manage PGx results, and concerns about privacy, cost, and equitable access were common. |
| Attitudes on pharmacogenetic testing in psychiatric patients with treatment-resistant depression. [29] | McCarthy et al. (2020), USA | Survey | Testing reveals that subjects were largely positive about the use of genetic testing to guide pharmacological treatment and help plan their future. Most subjects showed only modest concerns about the impact on family, inability to cope with the results, and fear of discrimination. The severity of depression did not predict the concern expressed about negative outcomes. |
| Scoping review of enablers and challenges of implementing pharmacogenomics testing in the primary care settings. [30] | Mai et al. (2024), various countries | Scoping review | Patients’ perspectives revealed limited awareness and understanding of pharmacogenomic testing, with concerns about privacy, potential misuse of genetic data, and fear of discrimination acting as key barriers. Anxiety over third-party access and uncertainty about the impact on future care reduced willingness to participate. Acceptance improved when information was clearly explained by trusted providers and testing was framed as part of routine, personalised care, but unclear communication and lack of public education often hindered engagement. |
| Understanding the Barriers and Enablers of Pharmacogenomic Testing in Primary Care: A Qualitative Systematic Review with Meta-Aggregation Synthesis. [31] | Qureshi et al. (2021), various countries | Systematic review | Patients showed general openness to pharmacogenomic testing, but engagement was limited by poor understanding of genetics, concerns about privacy and data misuse, and doubts about the test’s value, especially when communication was unclear. Lack of prior experience or visible benefits of personalized medicine further reduced trust. However, when PGx was integrated into care and clearly explained by providers, patients were more engaged, recognizing its value in improving medication safety and effectiveness. |
| Exploring perceptions, knowledge, and attitudes regarding pharmacogenetic testing in the medically underserved. [32] | Gawronski et al. (2023), USA | Survey | Only 21.1% of underserved respondents were aware of PGx testing, yet 60.6% showed interest when it was explained, and 75.8% supported its availability regardless of cost. Cost remained the top concern, reported by 52.7%, followed by fears about disease risk disclosure and insurance impact. |
| Assessment of patient perceptions of genomic testing to inform pharmacogenomic implementation. [33] | Ming Lee et al. (2017), USA | Qualitative study | Participants agreed that pharmacogenomics could inform prescribing and help identify problem prescriptions but expressed concerns over insurance coverage and employment discrimination. |
| Clinical Benefits and Utility of Pretherapeutic *DPYD* and *UGT1A1* Testing in Gastrointestinal Cancer. [34] | Roncato et al. (2024), Italy | Secondary analysis of a RTC | In the Italian cohort of the PREPARE trial, patients with gastrointestinal cancer treated with fluoropyrimidines and/or irinotecan who received pretreatment pharmacogenetic testing reported improved quality of life, regardless of whether they carried actionable DPYD or UGT1A1 variants. This benefit likely stemmed not only from clinical management but also from the psychological reassurance of having their genetic profile considered. The expectation of personalized care, even without dose changes, contributed to more positive treatment perceptions. An observed increase in quality-adjusted life-years in the intervention group supports this and aligns with evidence that pharmacogenomic testing enhances patient-reported outcomes and well-being. |

# **Supplementary Table 4. Collected evidence on organizational feasibility of pre-emptive PGx**

| **Title** | **Authors (Year), Country** | **Type of study** | **Key findings** |
| --- | --- | --- | --- |
| Pharmacogenetics in Italy: current landscape and future prospects. [35] | Floris et al. (2024), Italy | Report | Pharmacogenetic testing in Italy is gaining relevance, driven by international guidelines, increasing genotyping availability, and participation in large-scale studies. However, limited reimbursement and regulatory gaps continue to hinder its integration into clinical practice. Recent policy updates show progress but still exclude key tests, highlighting the need for national coordination and clearer implementation strategies. |
| Ten-year experience with pharmacogenetic testing for *DPYD* in a national cancer center in Italy: Lessons learned on the path to implementation. [36] | Bignucolo et al. (2023), Italy | Report | *DPYD* diagnostic activity at the center has greatly evolved over the years, shifting gradually from a post-toxicity to a pre-treatment approach. Development of pharmacogenetic guidelines by national and international consortia, genotyping, and IT technology evolution have impacted *DPYD* testing uptake in the clinics. Nationwide test reimbursement together with recommendations by regulatory agencies in Europe and Italy in 2020 definitely changed the clinical practice guidelines of fluoropyrimidines prescription. |
| Challenges and Opportunities in Implementing Pharmacogenetic Testing in Clinical Settings. [37] | Chang, W. C. et al (2021), Canada | Narrative review | While pharmacogenetic testing offers clear clinical benefits, its adoption is hindered by organizational limitations. Turnaround times are often incompatible with clinical workflows, and many health systems lack the infrastructure to process and integrate test results effectively. The absence of standardized procedures and coordinated implementation strategies across institutions contributes to fragmented efforts, reducing the impact and scalability of pharmacogenetic programs. |
| Barriers and facilitators for implementing a pharmacogenetic passport: lessons learned from reusing sequencing data. [38] | Roelofsen et al. (2025), Netherlands | Survey | Barriers to implementing a pharmacogenetic passport using reused sequencing data include fragmented IT systems requiring manual processing, limited pharmacogenetic knowledge among non-specialist clinicians, lack of clear guidelines and standard protocols, and unclear task distribution among healthcare professionals. Additional concerns involve unequal access for patients without prior sequencing data and low institutional prioritization, all of which hinder broader adoption. |
| Pharmacogenomics decision support in the U-PGx project: Results and advice from clinical implementation across seven European countries. [39] | Blagec et al. (2022), various countries | Implementation study | Implementing pharmacogenomics decision support tools across seven European countries proved feasible and was generally well received by healthcare providers. A total of 6,884 samples were genotyped, with 83.8% yielding at least one actionable phenotype, and reports were delivered with a median turnaround time of 20 minutes. Satisfaction with the tools was high, but adoption varied by country due to differences in infrastructure and user familiarity. Despite some delays, the project demonstrated strong potential for broader rollout, provided that regulatory, technical, and educational barriers are addressed. |
| Assessment of the pharmacogenomics educational environment in Southeast Europe. [40] | Pisanu et al. (2014),  Italy | Survey | While a large proportion of residents and physicians acknowledged the utility of pharmacogenetic tests (93% and 79%, respectively), only a small percentage (16% of physicians and 7% of residents) felt confident in selecting and interpreting these tests based on their training. |
| Educating the Next Generation of Pharmacogenomics Experts: Global Educational Needs and Concepts. [62] | Just et al. (2019), various countries | Survey | Pharmacogenomics education among healthcare professionals across eight countries revealed high interest but substantial gaps in applied knowledge and confidence. A total of 49 participants attended a European summer school as part of the U-PGx project, with the majority coming from academic settings. While PGx was perceived as clinically valuable, especially for drugs with high adverse reaction risks, its practical use remained limited due to uncertainty in test interpretation and lack of structured training. |
| Medical education in pharmacogenomics—results from a survey on pharmacogenetic knowledge in healthcare professionals within the European pharmacogenomics clinical implementation project Ubiquitous Pharmacogenomics (U-PGx). [41] | Just et al. (2017), various countries | Survey | Seven European countries showed strong interest in pharmacogenomics, but significant gaps in knowledge and confidence remain. In a U-PGx pre-implementation survey of 70 healthcare professionals, 84.3% deemed PGx relevant to their practice, yet 65.7% had not used it in the past year, mainly due to lack of knowledge (40.0%) and uncertainty in interpretation. While 85.8% felt familiar with pharmacology, only 51.4% were confident in interpreting PGx results. Moreover, 41.4% couldn’t identify drugs needing PGx testing, and 37.2% were unsure how to adjust therapy based on results. |
| Healthcare provider and patient perspectives on the implementation of pharmacogenetic-guided treatment in routine clinical practice. [42] | Kaur et al. (2024), various countries | Survey | 64% percent of healthcare providers reported some familiarity with pharmacogenetic testing, yet few had practical experience, only 4% had ordered a test and 3% had recommended one. The main barriers to implementation were limited access to testing (55%), lack of knowledge (48%), and difficulty interpreting results (35%). Despite these obstacles, 98% believed that point-of-care testing devices could increase adoption in routine practice. Additionally, 90% emphasized the need for comprehensive training, and nearly half highlighted the importance of user-friendly databases and faster testing tools to support clinical decisions. |
| Pharmacogenetic Information in Clinical Guidelines: The European Perspective. [43] | Swen et al. (2018), various countries | Narrative review | Surveys among pharmacists and physicians revealed broad acceptance of pharmacogenomics (PGx) as a concept, and patients showed willingness to participate in PGx implementation studies. However, actual clinical uptake remained limited, as healthcare professionals infrequently ordered or recommended PGx testing. A key organizational barrier identified was the lack of accessible guidelines to translate PGx test results into actionable clinical decisions. |
| A Scoping Review of Pharmacogenomic Educational Interventions to Improve Knowledge and Confidence. [44] | Soueid et al. (2024), various countries | Scoping review | Educational interventions led to an average 21% increase in knowledge, a 37% increase in confidence interpreting pharmacogenomic data, and improved confidence in communicating with patients (41%) and healthcare professionals (44%). Case-based learning, simulation, and the use of mock or self pharmacogenomic data were the most effective strategies. Interprofessional learning was associated with greater communication skill gains. Long-term retention was assessed in only two studies, with mixed results. Overall, interactive and applied approaches proved more effective than didactic methods alone. |

Steering Committee Consultation Questionnaire

To evaluate the potential impact of implementing the pharmacogenetic passport in Italy, our technical group is collecting data and information through scientific literature reviews and consultations with experts and stakeholders. As members of the Steering Committee, we kindly ask you to provide your opinions on some key points regarding the implementation of this approach in Italy. We have included a series of questions to guide the collection of your contributions. We kindly ask you to share your opinions on these topics, or if you have any materials or contacts that could help explore these aspects. Additionally, you can include free comments at the end of the document.

1. In your opinion, on which population should the pharmacogenomic panel be conducted (e.g., the entire population, individuals undergoing polypharmacotherapy, geriatric population, pediatric population, etc.)? And in what healthcare setting (e.g., primary care physicians, hospital admissions, etc.)?
2. Do you think further research initiatives are necessary to implement the passport in Italy?
3. Regarding the development of guidelines for the use of the panel, who should lead the process, and which professionals need to be involved? From an organizational perspective, what actions are necessary to implement the panel (e.g., laboratory capacity, equipment, personnel, data integration, etc.)?
4. Regarding training, do you think specific training activities are necessary to effectively implement the technology? If so, who should be targeted?
5. What do you consider the main barriers (clinical practice, organizational, economic, etc.) to implementing the passport in Italy?
6. Do you believe there are particularly vulnerable populations that could be positively or negatively impacted by the implementation of the passport?

# **References**

1. Swen, J. J., van der Wouden, C. H., Manson, L. E., Abdullah-Koolmees, H., Blagec, K., Blagus, T., Böhringer, S., Cambon-Thomsen, A., Cecchin, E., Cheung, K. C., Deneer, V. H., Dupui, M., Ingelman-Sundberg, M., Jonsson, S., Joefield-Roka, C., Just, K. S., Karlsson, M. O., Konta, L., Koopmann, R., Kriek, M., … Ubiquitous Pharmacogenomics Consortium (2023). A 12-gene pharmacogenetic panel to prevent adverse drug reactions: an open-label, multicentre, controlled, cluster-randomised crossover implementation study. *Lancet (London, England)*, *401*(10374), 347–356. <https://doi.org/10.1016/S0140-6736(22)01841-4>
2. Elliott, L. S., Henderson, J. C., Neradilek, M. B., Moyer, N. A., Ashcraft, K. C., & Thirumaran, R. K. (2017). Clinical impact of pharmacogenetic profiling with a clinical decision support tool in polypharmacy home health patients: A prospective pilot randomized controlled trial. *PloS one*, *12*(2), e0170905. <https://doi.org/10.1371/journal.pone.0170905>
3. Brixner, D., Biltaji, E., Bress, A., Unni, S., Ye, X., Mamiya, T., Ashcraft, K., & Biskupiak, J. (2016). The effect of pharmacogenetic profiling with a clinical decision support tool on healthcare resource utilization and estimated costs in the elderly exposed to polypharmacy. *Journal of medical economics*, *19*(3), 213–228. <https://doi.org/10.3111/13696998.2015.1110160>
4. Fagerness, J., Fonseca, E., Hess, G. P., Scott, R., Gardner, K. R., Koffler, M., Fava, M., Perlis, R., Brennan, F. X., & Lombard, J. (2014). Pharmacogenetic-guided psychiatric intervention associated with increased adherence and cost savings. *The American journal of managed care*, *20*(5), e146–e156.
5. Van Driest, S. L., Shi, Y., Bowton, E. A., Schildcrout, J. S., Peterson, J. F., Pulley, J., Denny, J. C., & Roden, D. M. (2014). Clinically actionable genotypes among 10,000 patients with preemptive pharmacogenomic testing. *Clinical pharmacology and therapeutics*, *95*(4), 423–431. <https://doi.org/10.1038/clpt.2013.229>
6. Hoffman, J. M. et al. PG4KDS: a model for the clinical implementation of pre-emptive pharmacogenetics. *Am. J. Med. Genet. C. Semin. Med. Genet.* 166c, 45–55 (2014).
7. Bielinski, S. J., Olson, J. E., Pathak, J., Weinshilboum, R. M., Wang, L., Lyke, K. J., Ryu, E., Targonski, P. V., Van Norstrand, M. D., Hathcock, M. A., Takahashi, P. Y., McCormick, J. B., Johnson, K. J., Maschke, K. J., Rohrer Vitek, C. R., Ellingson, M. S., Wieben, E. D., Farrugia, G., Morrisette, J. A., Kruckeberg, K. J., … Kullo, I. J. (2014). Preemptive genotyping for personalized medicine: design of the right drug, right dose, right time-using genomic data to individualize treatment protocol. *Mayo Clinic proceedings*, *89*(1), 25–33. <https://doi.org/10.1016/j.mayocp.2013.10.021>
8. Gottesman, O., Scott, S. A., Ellis, S. B., Overby, C. L., Ludtke, A., Hulot, J. S., Hall, J., Chatani, K., Myers, K., Kannry, J. L., & Bottinger, E. P. (2013). The CLIPMERGE PGx Program: clinical implementation of personalized medicine through electronic health records and genomics-pharmacogenomics. *Clinical pharmacology and therapeutics*, *94*(2), 214–217. <https://doi.org/10.1038/clpt.2013.72>
9. Fernandez, C. A., Smith, C., Yang, W., Lorier, R., Crews, K. R., Kornegay, N., Hicks, J. K., Stewart, C. F., Kawedia, J. D., Ramsey, L. B., Liu, C., Evans, W. E., Relling, M. V., & Broeckel, U. (2012). Concordance of DMET plus genotyping results with those of orthogonal genotyping methods. *Clinical pharmacology and therapeutics*, *92*(3), 360–365. <https://doi.org/10.1038/clpt.2012.95>
10. Johnson, J. A., Burkley, B. M., Langaee, T. Y., Clare-Salzler, M. J., Klein, T. E., & Altman, R. B. (2012). Implementing personalized medicine: development of a cost-effective customized pharmacogenetics genotyping array. *Clinical pharmacology and therapeutics*, *92*(4), 437–439. <https://doi.org/10.1038/clpt.2012.125>
11. Oetjens, M. T., Denny, J. C., Ritchie, M. D., Gillani, N. B., Richardson, D. M., Restrepo, N. A., Pulley, J. M., Dilks, H. H., Basford, M. A., Bowton, E., Masys, D. R., Wilke, R. A., Roden, D. M., & Crawford, D. C. (2013). Assessment of a pharmacogenomic marker panel in a polypharmacy population identified from electronic medical records. *Pharmacogenomics*, *14*(7), 735–744. <https://doi.org/10.2217/pgs.13.64>
12. Convertino, I., Salvadori, S., Pecori, A., Galiulo, M. T., Ferraro, S., Parrilli, M., Corona, T., Turchetti, G., Blandizzi, C., & Tuccori, M. (2019). Potential Direct Costs of Adverse Drug Events and Possible Cost Savings Achievable by their Prevention in Tuscany, Italy: A Model-Based Analysis. *Drug safety*, *42*(3), 427–444. <https://doi.org/10.1007/s40264-018-0737-0>
13. Plumpton, C. O., Pirmohamed, M., & Hughes, D. A. (2019). Cost-Effectiveness of Panel Tests for Multiple Pharmacogenes Associated With Adverse Drug Reactions: An Evaluation Framework. *Clinical pharmacology and therapeutics*, *105*(6), 1429–1438. <https://doi.org/10.1002/cpt.1312>
14. Bouttell, J., Heggie, R., Oien, K., Romaniuk, A., VanSteenhouse, H., von Delft, S., & Hawkins, N. (2022). Economic evaluation of genomic/genetic tests: a review and future directions. *International journal of technology assessment in health care*, *38*(1), e67. <https://doi.org/10.1017/S0266462322000484>
15. Turongkaravee, S., Jittikoon, J., Rochanathimoke, O., Boyd, K., Wu, O., & Chaikledkaew, U. (2021). Pharmacogenetic testing for adverse drug reaction prevention: systematic review of economic evaluations and the appraisal of quality matters for clinical practice and implementation. *BMC health services research*, *21*(1), 1042. <https://doi.org/10.1186/s12913-021-07025-8>
16. Plumpton, C.O., Roberts, D., Pirmohamed, M. *et al.* A Systematic Review of Economic Evaluations of Pharmacogenetic Testing for Prevention of Adverse Drug Reactions. *PharmacoEconomics* 34, 771–793 (2016). <https://doi.org/10.1007/s40273-016-0397-9>
17. Berm, E. J., Looff, M.d, Wilffert, B., Boersma, C., Annemans, L., Vegter, S., Boven, J. F., & Postma, M. J. (2016). Economic Evaluations of Pharmacogenetic and Pharmacogenomic Screening Tests: A Systematic Review. Second Update of the Literature. *PloS one*, *11*(1), e0146262. <https://doi.org/10.1371/journal.pone.0146262>
18. Morris, S. A., Alsaidi, A. T., Verbyla, A., Cruz, A., Macfarlane, C., Bauer, J., & Patel, J. N. (2022). Cost Effectiveness of Pharmacogenetic Testing for Drugs with Clinical Pharmacogenetics Implementation Consortium (CPIC) Guidelines: A Systematic Review. *Clinical pharmacology and therapeutics*, *112*(6), 1318–1328. <https://doi.org/10.1002/cpt.2754>
19. Wong, W. B., Carlson, J. J., Thariani, R., & Veenstra, D. L. (2010). Cost effectiveness of pharmacogenomics: a critical and systematic review. *PharmacoEconomics*, *28*(11), 1001–1013. <https://doi.org/10.2165/11537410-000000000-00000>
20. Plöthner, M., Ribbentrop, D., Hartman, J. P., & Frank, M. (2016). Cost-Effectiveness of Pharmacogenomic and Pharmacogenetic Test-Guided Personalized Therapies: A Systematic Review of the Approved Active Substances for Personalized Medicine in Germany. *Advances in therapy*, *33*(9), 1461–1480. <https://doi.org/10.1007/s12325-016-0376-8>
21. Fragoulakis, V., Koufaki, M. I., Mlinšek, G., Blagus, T., Klen, J., Patrinos, G. P., Dolžan, V., & Mitropoulou, C. (2025). Cost-utility analysis of pharmacogenomics-guided tacrolimus treatment of Slovenian patients undergoing kidney transplantation in the U-PGx PREPARE study. *The pharmacogenomics journal*, *25*(1-2), 6. <https://doi.org/10.1038/s41397-025-00365-2>
22. Fragoulakis, V., Koufaki, M. I., Joefield-Roka, C., Sunder-Plassmann, G., & Mitropoulou, C. (2024). Cost-utility analysis of pharmacogenomics-guided tacrolimus treatment in Austrian kidney transplant recipients participating in the U-PGx PREPARE study. *The pharmacogenomics journal*, *24*(2), 10. <https://doi.org/10.1038/s41397-024-00330-5>
23. Fragoulakis, V., Roncato, R., Bignucolo, A., Patrinos, G. P., Toffoli, G., Cecchin, E., & Mitropoulou, C. (2023). Cost-utility analysis and cross-country comparison of pharmacogenomics-guided treatment in colorectal cancer patients participating in the U-PGx PREPARE study. *Pharmacological research*, *197*, 106949. <https://doi.org/10.1016/j.phrs.2023.106949>
24. Koufaki, M. I., Fragoulakis, V., Díaz-Villamarín, X., Karamperis, K., Vozikis, A., Swen, J. J., Dávila-Fajardo, C. L., Vasileiou, K. Z., Patrinos, G. P., & Mitropoulou, C. (2023). Economic evaluation of pharmacogenomic-guided antiplatelet treatment in Spanish patients suffering from acute coronary syndrome participating in the U-PGx PREPARE study. *Human genomics*, *17*(1), 51. <https://doi.org/10.1186/s40246-023-00495-3>
25. McDermott, J. H., Sharma, V., Newman, W. G., Wilson, P., Payne, K., & Wright, S. (2024). Public preferences for pharmacogenetic testing in the NHS: Embedding a discrete choice experiment within service design to better meet user needs. *British journal of clinical pharmacology*, *90*(7), 1699–1710. <https://doi.org/10.1111/bcp.16058>
26. Norris, M., Dalton, R., Alam, B., Eddy, E., Nguyen, K. A., Cavallari, L. H., Sumfest, J., Wiisanen, K., & Cicali, E. J. (2023). Lessons from clinical implementation of a preemptive pharmacogenetic panel as part of a testing pilot program with an employer-sponsored medical plan. *Frontiers in genetics*, *14*, 1249003. <https://doi.org/10.3389/fgene.2023.1249003>
27. Kaur, G., & Nwabufo, C. K. (2024). Healthcare provider and patient perspectives on the implementation of pharmacogenetic-guided treatment in routine clinical practice. *Pharmacogenetics and genomics*, *34*(7), 236–245. <https://doi.org/10.1097/FPC.0000000000000541>
28. Frigon, M. P., Blackburn, M. È., Dubois-Bouchard, C., Gagnon, A. L., Tardif, S., & Tremblay, K. (2019). Pharmacogenetic testing in primary care practice: opinions of physicians, pharmacists and patients. *Pharmacogenomics*, *20*(8), 589–598. <https://doi.org/10.2217/pgs-2019-0004>
29. McCarthy, M. J., Chen, Y., Demodena, A., Fisher, E., Golshan, S., Suppes, T., & Kelsoe, J. R. (2020). Attitudes on pharmacogenetic testing in psychiatric patients with treatment-resistant depression. *Depression and anxiety*, *37*(9), 842–850. <https://doi.org/10.1002/da.23074>
30. Mai, C. W., Sridhar, S. B., Karattuthodi, M. S., Ganesan, P. M., Shareef, J., Lee, E. L., & Armani, K. (2024). Scoping review of enablers and challenges of implementing pharmacogenomics testing in the primary care settings. *BMJ open*, *14*(11), e087064. <https://doi.org/10.1136/bmjopen-2024-087064>
31. Qureshi, S., Latif, A., Condon, L., Akyea, R. K., Kai, J., & Qureshi, N. (2022). Understanding the barriers and enablers of pharmacogenomic testing in primary care: a qualitative systematic review with meta-aggregation synthesis. *Pharmacogenomics*, *23*(2), 135–154. <https://doi.org/10.2217/pgs-2021-0131>
32. Gawronski, B. E., Cicali, E. J., McDonough, C. W., Cottler, L. B., & Duarte, J. D. (2023). Exploring perceptions, knowledge, and attitudes regarding pharmacogenetic testing in the medically underserved. *Frontiers in genetics*, *13*, 1085994. <https://doi.org/10.3389/fgene.2022.1085994>
33. Lee, Y. M., McKillip, R. P., Borden, B. A., Klammer, C. E., Ratain, M. J., & O'Donnell, P. H. (2017). Assessment of patient perceptions of genomic testing to inform pharmacogenomic implementation. *Pharmacogenetics and genomics*, *27*(5), 179–189. <https://doi.org/10.1097/FPC.000000000000027>
34. Roncato, R., Bignucolo, A., Peruzzi, E., Montico, M., De Mattia, E., Foltran, L., Guardascione, M., D'Andrea, M., Favaretto, A., Puglisi, F., Swen, J. J., Guchelaar, H. J., Toffoli, G., & Cecchin, E. (2024). Clinical Benefits and Utility of Pretherapeutic DPYD and UGT1A1 Testing in Gastrointestinal Cancer: A Secondary Analysis of the PREPARE Randomized Clinical Trial. *JAMA network open*, *7*(12), e2449441. <https://doi.org/10.1001/jamanetworkopen.2024.49441>
35. Floris, M., Moschella, A., Alcalay, M., Montella, A., Tirelli, M., Fontana, L., Idda, M. L., Pharmacogenomics Working Group of the Italian Society of Human Genetics (SIGU), Guarnieri, P., Capasso, M., Mammì, C., Nicoletti, P., & Miozzo, M. (2024). Pharmacogenetics in Italy: current landscape and future prospects. *Human genomics*, *18*(1), 78. <https://doi.org/10.1186/s40246-024-00612-w>
36. Bignucolo, A., De Mattia, E., Roncato, R., Peruzzi, E., Scarabel, L., D'Andrea, M., Sartor, F., Toffoli, G., & Cecchin, E. (2023). Ten-year experience with pharmacogenetic testing for *DPYD* in a national cancer center in Italy: Lessons learned on the path to implementation. *Frontiers in pharmacology*, *14*, 1199462. <https://doi.org/10.3389/fphar.2023.1199462>
37. Chang, W. C., Tanoshima, R., Ross, C. J. D., & Carleton, B. C. (2021). Challenges and Opportunities in Implementing Pharmacogenetic Testing in Clinical Settings. *Annual review of pharmacology and toxicology*, *61*, 65–84. <https://doi.org/10.1146/annurev-pharmtox-030920-025745>
38. Roelofsen, A. H. A., Kreeftenberg, L. L., van El, C. G., Henneman, L., Rigter, T., Sie, D., Bet, P. M., & Cornel, M. C. (2025). Barriers and facilitators for implementing a pharmacogenetic passport: lessons learned from reusing sequencing data. *Pharmacogenomics*, 1–14. Advance online publication. <https://doi.org/10.1080/14622416.2025.2504862>
39. Blagec, K., Swen, J. J., Koopmann, R., Cheung, K. C., Crommentuijn-van Rhenen, M., Holsappel, I., Konta, L., Ott, S., Steinberger, D., Xu, H., Cecchin, E., Dolžan, V., Dávila-Fajardo, C. L., Patrinos, G. P., Sunder-Plassmann, G., Turner, R. M., Pirmohamed, M., Guchelaar, H. J., Samwald, M., & Ubiquitous Pharmacogenomics Consortium (2022). Pharmacogenomics decision support in the U-PGx project: Results and advice from clinical implementation across seven European countries. *PloS one*, *17*(6), e0268534. <https://doi.org/10.1371/journal.pone.0268534>
40. Pisanu, C., Tsermpini, E. E., Mavroidi, E., Katsila, T., Patrinos, G. P., & Squassina, A. (2014). Assessment of the pharmacogenomics educational environment in Southeast Europe. *Public health genomics*, *17*(5-6), 272–279. <https://doi.org/10.1159/000366461>
41. Just, Katja S et al. “Educating the Next Generation of Pharmacogenomics Experts: Global Educational Needs and Concepts.” *Clinical pharmacology and therapeutics* vol. 106,2 (2019): 313-316. doi:10.1002/cpt.1471
42. Just, K. S., Steffens, M., Swen, J. J., Patrinos, G. P., Guchelaar, H. J., & Stingl, J. C. (2017). Medical education in pharmacogenomics-results from a survey on pharmacogenetic knowledge in healthcare professionals within the European pharmacogenomics clinical implementation project Ubiquitous Pharmacogenomics (U-PGx). *European journal of clinical pharmacology*, *73*(10), 1247–1252. <https://doi.org/10.1007/s00228-017-2292-5>
43. Kaur, G., & Nwabufo, C. K. (2024). Healthcare provider and patient perspectives on the implementation of pharmacogenetic-guided treatment in routine clinical practice. *Pharmacogenetics and genomics*, *34*(7), 236–245. <https://doi.org/10.1097/FPC.0000000000000541>
44. Swen, J. J., Nijenhuis, M., van Rhenen, M., de Boer-Veger, N. J., Buunk, A. M., Houwink, E. J. F., Mulder, H., Rongen, G. A., van Schaik, R. H. N., van der Weide, J., Wilffert, B., Deneer, V. H. M., Guchelaar, H. J., & Dutch Pharmacogenetics Working Group (DPWG) of the Royal Dutch Pharmacists Association (KNMP) (2018). Pharmacogenetic Information in Clinical Guidelines: The European Perspective. *Clinical pharmacology and therapeutics*, *103*(5), 795–801. <https://doi.org/10.1002/cpt.1049>
45. Soueid, R., Michael, T. J. F., Cairns, R., Charles, K. A., & Stocker, S. L. (2024). A Scoping Review of Pharmacogenomic Educational Interventions to Improve Knowledge and Confidence. *American journal of pharmaceutical education*, *88*(3), 100668. <https://doi.org/10.1016/j.ajpe.2024.100668>
